# Career development of international medical graduates in Canada: status of the unmatched

**DOI:** 10.1057/s41599-023-01534-z

**Published:** 2023-01-30

**Authors:** Yiming Wang, Rajkumar Luke Vijendra Das, Tatiana Lapa, Peter Marosan, Rosemary Pawliuk, Heidi D. Chable, Deidre Lake, Aisha Lofters

**Affiliations:** 1 Medical Genetics and Genomics Residency Program, University of Toronto, Toronto, ON Canada; 2 Division of Clinical and Metabolic Genetics, The Hospital for Sick Children, Toronto, ON Canada; 3 Li Ka Shing Knowledge Institute, St. Michael’s Hospital, Toronto, ON Canada; 4 Association of International Physicians and Surgeons of Ontario, Toronto, ON Canada; 5 Department of Family Practice, University of British Columbia, Vancouver, BC Canada; 6 Society of Canadians Studying Medicine Abroad, Langley, BC Canada; 7 Association of International Medical Doctors of British Columbia, Vancouver, BC Canada; 8 Alberta International Medical Graduates Association, Calgary, AB Canada; 9 Women’s College Hospital, Toronto, ON Canada; 10 Department of Family & Community Medicine, University of Toronto, Toronto, ON Canada; 11 Dana Lana School of Public Health, University of Toronto, Toronto, ON Canada

**Keywords:** Education, Health humanities

## Abstract

With limited residency positions, the majority of international medical graduates living in Canada and other developed countries cannot work as physicians. The educational experience and career development of unmatched international medical graduates (those who are not matched to a residency position) residing in Canada have never been studied. Through an innovative collaboration of provincial international medical graduate organizations, we launched an online survey targeting the career development of unmatched international medical graduates, with 356 survey responses entering data analysis. Respondents reported that at the early career planning stage, close to a third had not had adequate knowledge of their career prospects in Canada. Although official resources are available, most respondents found that the information did not match well with reality. After arriving in Canada, educational resources for unmatched international medical graduates are scarce. The majority of them work in healthcare-related fields but reported significant difficulties finding these positions, and there were limited career training opportunities. Among respondents who were no longer pursuing residency positions and had moved on to alternate career paths, we found, unexpectedly, that 69% of them reported previous residency application experience did not contribute to their current occupation, and most were not satisfied with their current career status and continuing education opportunities. In conclusion, the unmatched international medical graduates could serve as a vital reservoir of skilled medical professionals to serve the community, especially during public health crises. Continuing education and career support of unmatched international medical graduates are crucial for their career development and should be an area of priority for policymakers. Career support, especially for alternative career paths, can be built on the current services that exist in most provinces in Canada. In addition, novel online and social media tools can be utilized to increase the outreach of these programs.

## Introduction

International medical graduates (IMGs) or immigrant physicians are a major source of medical staff in many developed countries (Nwadiuko et al., 2018; Parsi, 2008). In Canada, IMGs are physicians who graduated from medical schools outside of Canada and the United States, including immigrant IMGs (I-IMGs) and Canadians who studied medicine abroad (CSAs) (Mok et al., 2011). IMGs make up about one-quarter of Canada’s doctors, and for some provinces, this ratio is considerably higher—46% in Saskatchewan and 38% in Newfoundland and Labrador (Canadian Institute for Health Information CIHI, 2012). In Canada, IMGs can apply for a limited number of residency positions (around 300–400 positions each year) through the Canadian Resident Matching Service (CaRMS) (Canadian Resident Matching Service (CaRMS), 2021). Due to the limited residency spots, the chance of acquiring a postgraduate training position for most IMGs in Canada is slim (Grierson et al., 2017; Watts et al., 2011; Obara, 2012). In 2020, 418 IMGs were matched from 2085 IMG applicants registered with the CaRMS (Canadian Resident Matching Service (CaRMS), 2020), indicating that about 80% of IMGs (versus <3% for graduates from Canadian medical schools) could not enter residency training in Canada. IMGs residing in Canada have a significantly lower successful match rate than in other developed countries, such as the United States and Britain, which offers a unique opportunity to study their career development (Ranasinghe, 2015; Kehoe et al., 2016).

Having a clear career path is vital for the well-being of IMGs. Our study of IMGs in Ontario showed that they suffer from feelings of anger, shame, desperation, and regret due to the lack of educational support and the inability to advance their career in Canada (Lofters et al., 2014). The career development needs of most IMGs include two aspects: (1) preparing for licensing exams in Canada and integration into the culture of local clinical practice (Lockyer et al., 2007); (2) training for opportunities in alternative career paths beyond residency (Turin et al., 2021). There is a need for continuing education for IMGs, irrespective of their eventual career choice. Most IMGs matched to residency programs report significant difficulties navigating their initial residency months due to unfamiliarity with the knowledge and culture unique to practice in Canada (Najeeb et al., 2019). For IMGs exploring alternative career paths, a lack of skill training and bridging programs are key barriers to job hunting and career success (Turin et al., 2021). While many previous studies reported a lack of continuing education and career support services, there is no detailed understanding of the status of continuing education for IMGs, especially for new immigrants and unmatched IMGs (Neiterman et al., 2017; Turin et al., 2022).

It is vital to understand the career status of unmatched IMGs and prevent them from becoming victims of “brain waste”, and the underutilization of their medical skills (Pang et al., 2002; Lofters et al., 2013, 2014). In addition, the large number of unmatched IMGs currently residing in Canada and other developed countries is a vital reservoir of skilled medical professionals who can be mobilized during public health crises such as the COVID-19 pandemic (Elshazly et al., 2020; Gutman et al., 2021). For IMGs to take functional roles in the public health system, training and continuing medical education are required (Hashim, 2017). However, there is a gap in the current literature on career development, maintenance of medical skills, and the educational needs of unmatched IMGs. Also, it is unclear what resources are available to IMGs at the early planning stage before making the vital career decision to immigrate to Canada or leave Canada for international medical schools.

Thus, we conducted the International Medical Graduates-Career Development in Canada (IMG-CDC) study, which focuses on the career development of IMGs currently not practicing as physicians in Canada. Through an online survey, we received feedback from IMGs in Canada on the access to information and resources at the early career planning stage, and the education and career support received after immigrating to Canada or returning from international medical schools. Uniquely, we captured data on unmatched IMGs who are no longer actively pursuing a residency position and have moved on to an alternative career path. The IMG-CDC study also represents the first collaborative effort of IMG organizations across Canada to guide the allocation of government and social resources in assisting IMGs.

## Methods

### Recruitment strategy

To maximize the scope of this study, we aim to recruit IMGs across Canada rather than limiting the study to a particular province. This study is the first research project conducted jointly by IMG organizations across Canada. As email communication is the primary communication method between IMG organizations with their members, potential survey participants were contacted by email (see Supplementary material) via the mailing lists of the Association of International Physicians and Surgeons of Ontario (AIPSO) (Wang et al., 2018), Society of Canadians Studying Medicine Abroad (SOCASMA), Association of International Medical Doctors of British Columbia (AIMDBC), and Alberta International Medical Graduates Association (AIMGA). The invitation email was also posted on the Telegram group of AMCA (a local private provider of Medical Council of Canada exams preparation services). The email contained a link to the online survey with informed consent, and participation was voluntary. The survey’s mailing list was created from IMGs registered with the above organizations in the past 10–20 years. The purpose of including historical and potentially inactive email addresses was to capture IMGs who have established themselves in an alternative career path, although this design would inevitably decrease our survey response rate. This approach ensures that we can probe the long-term career development of IMGs. In total, ~6000 emails were sent. The participants included two main categories of currently unmatched IMGs: (1) IMGs who are actively preparing for residency application in Canada; (2) IMGs who attempted residency application in the past but are no longer actively preparing for residency application. We also expanded our study population to a third group of IMGs who have been successfully matched to (or already completed) residency programs in Canada or the USA, to explore the difference in the educational support received between the matched and unmatched IMGs. There was also a small group of respondents who had not begun residency applications or were undecided. The study received approval from the St. Michael’s Hospital research ethics board.

### Survey

The survey was housed on Qualtrics (Qualtrics®XM) through the Department of Family & Community Medicine, University of Toronto. The questionnaire (see Supplementary material) contained five sections that targeted the current literature gap on IMGs in Canada: (1) Basic demographics of the participants; (2) Early-stage career planning; (3) Continuing education and training; (4) Current career development status; and (5) Questions specific to each of the three groups of IMGs mentioned above. Most questions were close-ended, but open-ended questions were included to allow participants to describe their individual career development experience and educational needs. The questionnaire has 9 pages/screens with less than 10 questions per page. It took ~10–15 min to complete. The survey was pretested for clarity, the relevance of questions, and completion time with physicians at the University of Toronto and IMGs at AIPSO. The pretest feedback helped modify the questionnaire and increased the clarity and efficiency for IMG participants and potential future knowledge users. Due to the participating IMG organizations in different provinces sending out the recruitment email at staggered time points, the survey was opened for one year (from June 2018 to July 2019), and 476 survey responses were collected.

### Analysis

Of the 476 responses with a unique IP address, those that were missing I-IMG/CSA status, province of residence, or classification (actively preparing for application, no longer planning to apply, or successfully matched) were excluded, leaving 356 participants. On average, participants completed 81.9% of the questionnaire, with 266 participants completing the survey in its entirety. Data analysis was conducted using Qualtrics Survey Software and Excel. Chi-square, non-parametric one-way ANOVA, or two-tailed non-parametric *t*-tests were used for statistical analysis of quantitative data. Ratios were calculated from the number of respondents with a specific answer divided by the number of total respondents to that question. Open-ended questions were analyzed by two of the authors independently (YW and RLVD), and the recurring themes were summarized. Representative responses to open-ended questions are quoted in their original form, with only minor grammatical corrections for clarity. The Basic demographics of the participants are summarized in Table 1.

**Table 1:** Basic demographics of study participants.

Data group	Subcategory	Number	Percentage
Total respondents		476	
Included in analysis		356	100.0
Age (years)	29 or younger	70	19.7
	30–39	154	43.3
	40–49	88	24.7
	50+	44	12.4
Gender	Male	128	36.0
	Female	228	64.0
Region of Medical School Attended	Africa	50	14.0
	Asia-Middle east	67	18.8
	Asia-South	83	23.3
	Asia-Other	25	7.0
	Caribbean	33	9.3
	Europe	68	19.1
	Central and South America	27	7.6
	Oceania	3	0.8
Province of Residence	Ontario	167	46.9
	Alberta	109	30.6
	British Columbia	64	18.0
	Manitoba	5	1.4
	Quebec	5	1.4
	Saskatchewan	4	1.1
	Nova Scotia	1	0.3
	Northwest Territories	1	0.3
CSA or I-IMG	CSA	88	24.7
	I-IMG	268	75.3
Current career status	Unmatched-actively preparing for resident match	211	59.3
	Unmatched-not active	71	19.9
	Matched	58	16.3
	Other (not started application or undecided)	16	4.5

## Results

### Demographics of survey respondents

The respondents show a predominance of female (228/356, 64.0%) and aged over 30 (286/356, 80.3%), which is similar to the largest Canadian IMG dataset (*n* = 876, female 58.3%, age >30, 78.4%) reported based on family physicians from the Canadian IMG database (Mathews et al., 2017). Our finding of the leading IMG country of origin (e.g., South Asian countries) is also consistent with previous reports on IMG demographics (Lofters et al., 2013; Yen et al., 2016).

To enable the capture of IMGs no longer actively pursuing a residency position and in an alternative career path, we included historical and potentially inactive email addresses, resulting in a lower response rate, although the actual response rate may be significantly higher. Based on the approximate 6000 emails sent, we have a total response rate of 7.9% (476/6000). The response rate for the current active AIPSO members who regularly joined our educational activities was much higher at 70% (80/114).

Unsurprisingly, the largest group of respondents are IMGs currently applying for residency (59.3%) and are likely active members of the participating IMG organizations (Table 1). There was also significant participation from those not actively applying for residency (19.9%) and those matched to a residency program (16.3%).

### The early career planning stage

We define the early career planning stage as the career decision-making stage prior to immigrating to Canada for I-IMGs or before leaving Canada for international medical schools for CSAs. Surprisingly, close to a third of IMGs reported that they had never heard of the licensing examinations and details of the residency application process in Canada at this stage. This number was significantly higher in unmatched I-IMGs (71/198, 35.9%) as compared to unmatched CSAs (6/38, 15.8%) and matched IMGs (7/52, 13.9%) (*p* < 0.05) (Table 2).

**Table 2:** Information available at the early career planning stage.

		Unmatched % (Number)		Matched % (Number)	Total % (Number)
		I-IMG	CSA		
Knowledge of licensing exams and residency application process	Never heard of these info (before arriving in Canada or leaving Canada for international schools)	35.9 (71/198)	15.8 (6/38)	13.9 (7/52)	29.2 (84/288)
Source of Knowledge before and after arriving in or returning to Canada	MCC website	73.7 (146/198)	86.8 (33/38)	76.9 (40/52)	76.0 (219/288)
	CaRMS website	19.7 (39/198)	39.5 (15/38)	44.2 (23/52)	26.7 (77/288)
	Physiciansapply website	37.4 (74/198)	42.1 (16/38)	46.2 (24/52)	39.6 (114/288)
	Canadian embassy/consulate	1.0 (2/198)	0.0 (0/38)	5.8 (3/52)	1.7 (5/288)
	Canadian physicians	7.1 (14/198)	5.3 (2/38)	11.5 (6/52)	7.6 (22/288)
	Friends and family members	46.0 (91/198)	36.8 (14/38)	51.9 (27/52)	45.8 (132/288)
	Other	12.6 (25/198)	15.8 (6/38)	25.0 (13/52)	15.3 (44/288)

**Fig. 1:**
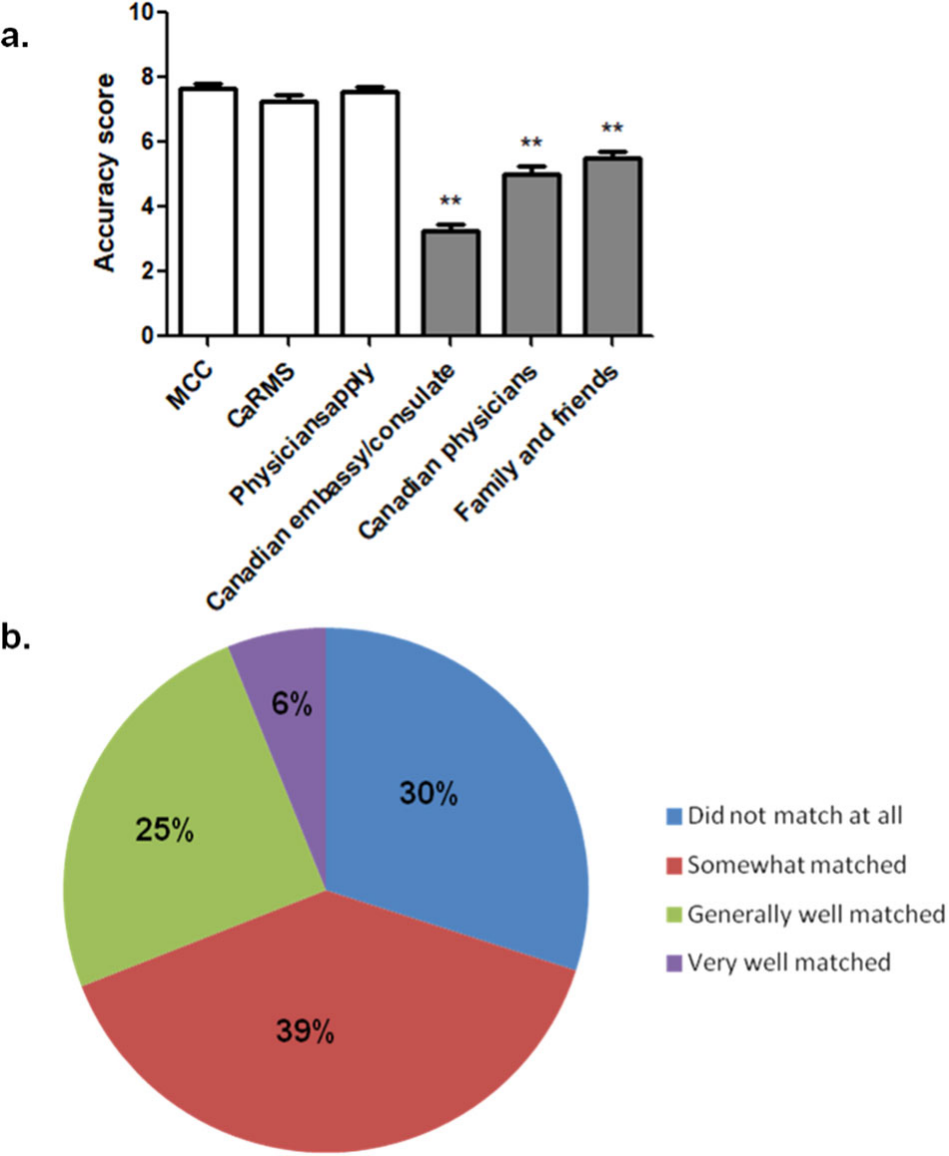
Accuracy of information available at the early career planning stage. **P < 0.01. **a** Acurracy of different resources. Scores were provided on a scale from 1 (the least accurate) to 10 (the most accurate). One-way ANOVA and non-parametric *t*-test showed significantly lower scores received by the Canadian embassy/consulate, Canadian physicians, and family and friends compared to each of the top three choices (MCC, CaRMS, Physiciansapply). Error bars indicate the standard error of the mean (SEM). **b** match between information received at the early career planning stage and the career reality in Canada.

All IMGs ranked the Medical Council of Canada (MCC) website as their top source of information, followed by the Physiciansapply and CaRMS websites (Table 2). IMG support organizations such as Health Force Ontario (HFO), AIPSO, AIMGA, AIMDBC, and SOCASMA were also reported as helpful. Among these resources, MCC, Physiciansapply, and CaRMS websites were considered by IMGs to be the most accurate. However, other information sources received significantly lower scores, with the Canadian embassy and consulate receiving the lowest ranking for accuracy (Fig. 1a). Many I-IMGs reported that while their medical training and experience were highly valued during the immigration process, they did not receive any specific information on their career path from Canadian embassies and consulates.

The majority of IMGs found the information available did not match well with reality (Fig. 1b). Almost all IMGs agreed that MCC, Physiciansapply, and CaRMS websites provide accurate, up-to-date guidance on the examinations and matching process. However, they reported that these websites do not provide sufficient information on the competitiveness of residency matching in Canada, which is crucial for decision-making at the early career planning stage (i.e., whether to immigrate to Canada or attend international medical schools). Most IMGs mentioned not realizing the difficulties in attaining a residency position and the complexity of the application process:

- I realized that there is no guarantee of getting matched and practicing as a doctor. (I-IMG, 40–49 y/o, female, unmatched)
- I … thought that the examinations to apply back to Canada would be easy to complete when I had graduated, but I was not aware of how difficult the examinations were and how competitive the environment is for CSAs. (CSA, < 29 y/o, female, unmatched)
- All hidden expectations, getting reference letters, needs for networking, which are difficult to meet… (I-IMG, > 50 y/o, male, unmatched)

### Educational resources for residency application

Overall, most IMGs (223/282, 79%) felt they do not have enough support for exam preparation and residency application, which is a long and costly process. The majority of IMGs reported actively trying for residency for between 1 and 10 years and spending over $10,000 in the process. Most IMGs had participated in some educational activities, with different participation in provincial support centers (110/282, 39%), commercial courses (149/289, 52%), and IMG organizations/study groups (218/289, 75%). All of these resources received a positive rating for their service (>5 on a scale of 0–10). As for which education resource is most valuable, IMG organizations (e.g., AIMDBC, AIMGA, AIPSO, SOCASMA) and local study groups received significantly higher satisfaction scores (7.10 ± 0.14, versus 6.16 ± 0.20 for commercial courses and 5.66 ± 0.22 for provincial support centers, *P* < 0.01). The common themes of IMG organizations and study groups include a sense of community, peer support, and low cost:

- It is good to join activities with people on the same page as you. It keeps you motivated and focused. (I-IMG, < 29 y/o, female, unmatched)
- IMGs keep encouraging each other and, moving forward, share personal experiences with exam preparation… free of charge. (I-IMG, 30–39 y/o, female, unmatched)
- AIMGA is helping a lot to attain a residency position and gives many opportunities like arranging observerships, IELTS classes, etc. (I-IMG, 30–39 y/o, female, matched)

The model of government support through an IMG organization exemplified by AIMGA was universally acknowledged as highly effective. AIMGA is unique in Canada as it is governed by a board of IMGs and federally funded, while IMG organizations in other provinces suffer from a shortage of funding.

Our data also revealed that matched IMGs are an excellent resource for delivering educational support for unmatched IMGs. The majority of matched IMGs (35/40, 88%) expressed willingness to help unmatched IMGs, and 77% (30/39) of them were actively participating in IMG education and training activities as volunteers. These activities included giving lectures, arranging clinical observerships, offering career guidance, and editing resumes/job applications.

### Educational resources for alternative career paths

Almost half (110/207, 53%) of all IMGs were currently employed in alternative careers—defined as employment other than medical practice in Canada or the USA. The employment rates were similar for I-IMGs and CSAs (97/181, 54% vs. 13/26, 50%). Among those in alternate careers, the majority (75/110, 68%) reported working in a healthcare-related field. These included various positions such as medical office assistants, administrators, research positions, care aides, nurses, and pharmacy assistants (Table 3).

**Table 3:** Alternate careers in healthcare and sources of alternative employment.

Alternative career	Number	Percentage
Allied health—Nursing, Pharmacy assistant, Teaching, Technicians	18	24
Clinical assistants	17	22.6
Medical office assistants, administrators	14	18.6
Research coordinator, assistants, other positions	13	17.3
Health care aide	7	9.3
Other health care	6	8
*Total*	75	100
**Source of employment info**		
Employment advertisement online	36	29.7
Family members or friends	37	30.5
Government IMG support programs	11	9.0
Training in an educational institution (university, college, etc.)	7	5.7
Other	30	24.8
*Total*	121	100

Most IMGs working in alternate healthcare positions reported finding these positions through online job searches or from family and friends (Table 3). However, there was little access to career-related training or education resources. Of all currently employed respondents, less than 10% (11/121) of IMGs utilized educational, or career support services of government IMG support centers. Only around 6% (7/121) of respondents reported receiving formal career training in an Canadian educational institution such as a university or college.

### The educational experience for the unmatched IMGs who are no longer actively applying

When asked why they stopped pursuing a residency, the top three reasons given by this group of IMGs were frustration (44/70, 63%), financial strain (37/70, 53%), and family responsibilities (28/70, 40%). Surprisingly, more than two-thirds of unmatched IMGs in this category reported that their previous experience of licensing exams and residency applications did not contribute to their current occupation at all (69% (27/39) giving a score of 1 when asked to rank the helpfulness from 1 to 10). Most IMGs in this group (27/37, 73%) had received some educational support for their career development. These included professional training at college, graduate studies, English as a second language (ESL) classes, academic conferences, and open hospital/university lectures. However, most IMGs were not satisfied with their current career status (20/33, 61%) and expressed a desire to see more government and community support for their alternative career development (42/45, 93%). Overall, IMGs in this category reported wanting more financial and educational support:

- The government could reimburse courses that lead to an alternative career. (I-IMG, 40–49 y/o, female, unmatched)
- … Financial support, such as OSAP (Ontario Student Assistance Program). (I-IMG, 30–39 y/o, male, unmatched)
- Work with the hospital authorities to create more opportunities for IMGs. This will deal with IMGs’ unhappiness/underemployment and inadequate healthcare for the community. (I-IMG, 30–39 y/o, female, unmatched)
- … need alternative, affordable educational opportunities in the field of healthcare (e.g., nursing, physical therapy, addiction and mental health, disability sectors, etc.). (I-IMG, 40–49 y/o, male, unmatched)

Many IMGs reported that while government services to direct IMGs to alternative career paths exist in most provinces, there are few structured affordable educational resources or funding opportunities for IMGs to pursue these paths. Many IMGs suggested government-funded bridging programs to help IMGs acquire skills and social connections for alternative careers. With the difficulties in securing a satisfactory alternative career, 33% (14/43) of I-IMGs were considering leaving Canada and going back to their country of origin.

## Discussion

Our study is the first in Canada to systemically analyze the career development status and educational needs of unmatched IMGs. The unique collaboration of IMG organizations across Canada enabled us to capture a wide array of IMGs at different stages of career development. Importantly, we reached out to IMGs no longer actively pursuing residency applications, and whose career development has never been studied before.

At the early career development stage, it is vital for IMGs to have accurate, up-to-date information for making life-changing decisions to immigrate to Canada or leave for international medical schools. However, our data revealed that most IMGs did not have sufficient information to make informed career choices and were later frustrated by the unexpected difficulties of the residency matching process. These issues can be resolved by offering easily accessible and unambiguous data on the competitiveness of residency positions through websites such as MCC, Physiciansapply, and CaRMS. While these websites currently provide highly accurate information on examinations and the application process, there are no dedicated sections geared toward IMGs at the early career planning stage. As the primary contact for immigrants before landing, Canadian embassies and consulates in major immigrant-origin countries should also offer some guidance to I-IMGs on their career prospects and direct them to high-quality online resources.

After arriving in Canada or returning from international medical schools, most IMGs felt they did not have sufficient educational support. Our survey showed that IMGs value a sense of community and peer support and educational programs that are delivered by provincial and local IMG organizations at a low cost. The model of government funding of IMG organizations (e.g., AIMGA) is especially favorable, as this model ensures peer support and relieves the financial burden of attending private commercial courses. The government-funded IMG organizations also serve as registries of local IMGs and would provide valuable resources during public health crises when the mobilization of IMGs could be required. Notably, matched IMG residents and physicians in our survey reported eagerness to support IMG education, suggesting that this group can be an essential resource to offer continuing education and career guidance.

Our study identified significant under-utilization of government IMG support services in searching for alternative careers. Almost all IMGs reported significant difficulties in finding alternative career opportunities in healthcare, and many are forced to work in jobs unrelated to their medical training. However, very few IMGs (10%) turned to government organizations for career advice. Based on our personal communications, many IMGs are unaware of these services, while others have doubts about their effectiveness. We believe government IMG support centers should employ innovative strategies (e.g., social media) to increase their outreach and attract more IMGs to their existing alternative career programs.

For the first time (that we are aware of) in the Canadian literature, we obtained data on unmatched IMGs who are no longer actively applying for residency positions. Most of them were not satisfied with their career status and would like to see more funding and educational resources for their alternative career path. Surprisingly, most unmatched IMGs did not value the experience of their previous residency application process as helpful for their current occupation, suggesting that the educational needs for alternative career paths are different from those required for residency. We believe government investment in bridging programs for alternative careers is required. While some bridging programs are emerging in big cities, they are costly, and their outreach is limited. The web-based long-distance education network, widely used during the COVID-19 pandemic, can be applied to ensure reaching beyond major population centers at a low cost.

Our survey findings extended the previous data on IMGs in Canada from our group and the others. As previously suspected (Lofters et al., 2014), we confirmed with a large dataset that most IMGs did not have a clear understanding of the residency application process before immigrating to Canada or leaving for international medical schools. We also showed that the lack of access to alternative career services and continuing education opportunities existed across multiple provinces in Canada, consistent with previous reports in individual provinces (Neiterman et al., 2017; Turin et al., 2021, 2022). Significantly, this report contributed to narrowing the gap of literature on IMGs in Canada as previously identified (Neiterman et al., 2017; Turin et al., 2021). In contrast to previous literature focusing on the educational activities for residency application and clinical performance of IMGs (Lockyer et al., 2007; Mathews et al., 2017; Najeeb et al., 2019), we found that the educational needs for alternative career paths were different from those for residency application and licensing examinations. The experience of exam preparations did not contribute to the success of establishing alternative career paths for most IMGs. On the other hand, there needs to be more awareness of government-funded career support service, which usually provides official guides and facilitates access to alternative career opportunities. It is vital to incorporate a strategy to increase the outreach of the IMG programs, as more government agencies and local community-based organizations are involved in alternative career support for IMGs (Sikdar et al., 2022).

## Limitations

Although the IMG-CDC study has many strengths, including its multi-provincial nature, it was limited by incomplete coverage in some provinces, especially in provinces without a dedicated IMG organization. Efforts were made by the authors of this paper and others to advocate for the participation of government agencies in this survey without success. We hope this study can facilitate future investigations for the career development of IMGs across all provinces of Canada.

A large number of survey invitation emails were sent to capture IMGs no longer actively pursuing a residency position and in alternative career paths, whose last contact with us was over 5–10 years, or even 20 years ago. This study design resulted in a relatively low response rate, likely due to the high mobility of the IMG population and reduced interest in residency applications following many years of failure. However, the survey response rate of the current active members (70% for AIPSO) is much higher, and the key participant demographics are similar to the previously published datasets. Still, we cannot exclude the possibility that the respondents do not well represent the entire Canadian IMG population. Future studies, ideally conducted at the provincial or federal government level, may ensure the capture of a sizeable IMG dataset and provide validation to our conclusions. Nevertheless, based on the participating IMG organizations’ long-term interactions with IMGs, we believe the issues identified by the respondents of this study are those experienced by most Canadian IMGs.

## Conclusion

In Canada, unmatched IMGs could serve as a vital reservoir of skilled medical professionals to serve the community, especially during public health crises. However, many unmatched IMGs are in jobs that underutilize their skills. Our study showed that the vast majority of IMGs do not have sufficient education and career support after arriving in Canada, both for the preparation of licensing examinations and alternative career paths. Importantly, we identified that the educational needs for alternative career paths are distinct from residency applications, and most IMGs were not satisfied with their current career status and continuing education opportunities. Continuing education and career support of unmatched IMGs are crucial for their career development and should be an area of priority for policymakers. We believe the best approach to deliver these services is through a collaboration between government agencies and the local IMG organizations, as in the case of AIMGA. Career support, especially for alternative career paths, can be built on the current IMG services that exist in most provinces in Canada. In addition, novel online and social media tools can be utilized to increase the outreach of these programs.

## Supplementary information

Email to participants and questionnaire

## Data Availability

The datasets generated during and/or analyzed during the current study are available from the corresponding author upon reasonable request.
