# Supplementary material for: Career development of international medical graduates in Canada: status of the unmatched
Source: Humanit Soc Sci Commun. 2023 Jan 30;10(1):38. doi: 10.1057/s41599-023-01534-z (PMC9885407; doi:10.1057/s41599-023-01534-z)
Supplement: Supplementary file 1 — Email to participants and questionnaire [file 41599_2023_1534_MOESM1_ESM.pdf]

Dear Doctor,

Re: International Medical Graduates' Career Development in Canada (IMG CDC): A Cross-sectional Online Survey Study

This email serves to introduce the above-captioned research project; a collaborative study led by Dr. Yiming Wang (Association of International Physicians and Surgeons of Ontario and St. Michael's Hospital Department of Laboratory Medicine) and Dr. Aisha Lofters (St. Michael's Hospital Department of Family & Community Medicine). The purpose of this research study is to explore the challenges faced by IMGs for their career development in Canada and the potential solutions. This study represents the first research project in Canada initiated by an organization of IMGs like you (i.e., Association of International Physicians and Surgeons of Ontario (AIPSO)). By taking 10-15 minutes to complete the questionnaire, you will assist us in collecting data to advocate more support for IMGs in Canada. Please note that St. Michael's Hospital is not responsible for the contents of the survey.

Your consent to participate in this study is demonstrated by your voluntary completion and submission of the survey. If you decide to participate, you can change your mind at any time while completing the survey. However, there will be no option to withdraw data once the questionnaire has been submitted, as there are no identifiers on the survey itself. If you decide to participate, please complete all questions to the best of your ability. If any question on the survey is not applicable to you or you feel uncomfortable answering, please feel free to skip this question. Although we would appreciate your responses to all questions, none of the individual questions are mandatory. The responses will be completely anonymous (i.e. there is no way to link your name to the responses). The survey responses will only be seen by the study team.

Completed surveys will be reviewed by the study team for data entry and analysis. The results of this study may be presented at conferences, seminars or other public forums, and published in journals, but no information will be used in these presentations that would disclose your identity as a study participant.

Please do not hesitate to contact Dr. Yiming Wang and Dr. Aisha Lofters (contact information provided below) if you have any questions. If you have any concerns regarding your rights as a research participant, you may contact Dr. David Mazer, Chair, St. Michael's Hospital, Research Ethics Board at 416-864-6060 ext. 2557 during business hours.

Please print a copy of this document for your records.

Sincerely,

Yiming Wang MD PhD
Association of International Physicians
and Surgeons of Ontario (AIPSO)
Department of Laboratory Medicine
St. Michael's Hospital
416-864-6060 ext 77590

Aisha Lofters MD PhD CCFP
Dept of Family & Community Medicine
St. Michael's Hospital, Toronto, ON
416-867-7428

International Medical Graduates' Career Development in Canada (IMG CDC): A Cross-sectional Online Survey Study

Survey Questionnaire

Part I. Basic information

1. Which of the following best describes your current situation?
    - Actively preparing for residency application in Canada
    - Successfully matched to (or already finished) a residency program in Canada
    - Successfully matched to (or already finished) a residency program in the US
    - Attempted in the past, but no longer actively preparing for residency application in Canada
    - Other, please specify:

2. What is your age?
    - 29 or younger
    - 30-39
    - 40-49
    - 50+

3. What is your gender?
    - Male
    - Female

4. In which country did you attend high school?

5. In which country did you complete medical school?

6. What was your highest level of practice before you came to Canada (or returned to Canada if you are a Canadian who studied medicine abroad)?
    - Medical student
    - Resident
    - Attending physician
    - Consultant physician
    - General practitioner
    - Division head
    - Department head
    - Other, please specify:

7. What was your specialty before you came to Canada?

8. What was your academic ranking before arriving in Canada?

- Not applicable
- Lecturer
- Assistant professor
- Associate professor
- Full professor

9. In what year did you arrive in Canada (or returned to Canada if you are a Canadian who studied medicine abroad)?

10. In which province or territory do you currently live?

- Ontario
- Alberta
- British Columbia
- Manitoba
- New Brunswick
- Newfoundland & Labrador
- Nova Scotia
- Northwest Territories
- Nunavut
- Prince Edward Island
- Quebec
- Saskatchewan
- Yukon

Part II. Examination and Residency Application

1.
a. If you are not currently in a residency program, for how long have you been trying to obtain a residency spot in Canada (start from the time that you were seriously considering or preparing for exams and applications)?

- Less than 6 months
- 6 months to 1 year
- 1-2 years
- 2-5 years
- 5-10 years
- More than 10 years
- Not applicable

b. If you are currently in a residency program, for how long were you trying before you obtained a residency spot in Canada?

- Less than 6 months
- 6 months to 1 year
- 1-2 years
- 2-5 years
- 5-10 years
- More than 10 years
- Not applicable

2. When did you first hear of the licensing examinations and residency application process in Canada?

- Before arriving in Canada
- Before leaving Canada for medical school in a foreign country (for Canadians who studied abroad)
- After arriving in Canada

3. From what resources did you first acquire information regarding licensing examinations and residency application in Canada? (Please select all that apply)

- Medical Council of Canada (MCC) website
- Canadian Resident Matching Service (CaRMS) website
- Physiciansapply website
- Canadian embassy/consulate
- Canadain physicians
- Friends and family members in Canada
- Other, Please specify:

4. How accurate do you think is the information available to you regarding licensing examinations and residency application in Canada? For each item you choose in question 3, please rate the accuracy from 1-10 (1 represents not accurate, and 10 represents very accurate).

- MCC website
- CaRMS website
- Physiciansapply website
- Canadian embassy/consulate
- Canadian physicians
- Friends and family members in Canada
- Other, Please specify:

For each item you rate above, why do you think it is accurate or not accurate?

- MCC website:
- CaRMS website:
- Physiciansapply website:
- Canadian embassy/consulate:
- Canadain physicians:
- Friends and family members in Canada:
- Other, Please specify:

5. After arriving in or returning to Canada, how did your impression match with the reality of examinations /residency application process?

- Very well matched
- Generally well matched
- Somewhat matched
- Did not match at all

Please give the reasons for your rating above:

6. Which of the following exams have you passed already? (Please select all exams you have passed)

- Medical Council of Canada Evaluating Exam (MCCEE)
- National Assessment Collaboration Objective Structured Clinical Examination (NAC OSCE)
- Medical Council of Canada Qualifying Examination Part 1 (MCCQE 1)
- Medical Council of Canada Qualifying Examination Part 2 (MCCQE 2)
- United States Medical Licensing Examination (USMLE) Step 1
- USMLE Step 2 Clinical Knowledge (CK)
- USMLE Step 2 Clinical Skills (CS)
- USMLE Step 3

7. Approximately how much money have you spent in the course of applying for a residency position (e.g., exams, courses, applications)?

- Less than 1,000
- $1,000-$5,000
- $5,000-$10,000
- $10,000-$15,000
- More than $15,000

8. Have you been registered with any provincial IMG career support centers (such as Practice Ready Assessment Programs in some provinces or similar government funded organizations in other provinces)?

- Yes
- No

If yes, please rate your experience from 1-10 (1 represents the worst experience, and 10 represents the best experience):
Please give the reasons for your rating above:

9. Have you taken a commercial course for exam preparation?

- Yes
- No

If yes, please rate your experience from 1-10 (1 represents the worst experience, and 10 represents the best experience):
Please give the reasons for your rating above:

10. Have you joined activities of any IMG organizations or study groups?

- Yes
- No

If yes, please rate your experience from 1-10 (1 represents the worst experience, and 10 represents the best experience):
Please give the reasons for your rating above:

11. Do you think you receive enough support for examinations and residency application in Canada?

- Yes
- No

12. What improvements in the IMG support system (e.g., government programs, IMG organizations, commercial courses) would you like to see?

Part III. Continuing Education and Training

1. Have you traveled back to your country of origin to practice medicine since you immigrated to Canada?
- o Yes.
- o No, but considering doing this in the near future.
- o No, never considered this option.
- o Not applicable.

If yes, how long did you practice there?
- o Less than 6 months
- o 6 months to 1 year
- o 1-2 years
- o 2-5 years
- o 5-10 years
- o More than 10 years

2. Do you have access to any continuing education or training opportunities in your specialty in Canada?
- o Yes.
- o No.

If you answered yes above, please provide your answer to question 3:
3. What kind of continuing education or training opportunities have you had?
- o Clinical fellowship
- o Clinical observership
- o Research fellowship
- o Graduate study
- o Professional training at a college
- o Conferences
- o Continuing Medical Education (CME) lectures and seminars
- o Hospital/University open lectures/grand rounds
- o Other, please specify:

4. From a scale of 1-10 (1 represents least difficult, and 10 represents extreme difficult), how difficult do you feel it is to maintain your professional knowledge and skills in Canada before matching to a residency program?

5. What kind of continuing education or training opportunities do you consider most important for you?
- o Clinical fellowship

- Clinical observership
- Research fellowship
- Graduate study
- Professional training at a college
- Conferences
- Hospital/University open lectures/grand rounds
- Other, please specify

6. How do you think government IMG support programs and IMG organizations can develop continuing education or training opportunities for IMGs?

7. Do you find your language skills (English or French) to be a major factor that affects your access to continuing education or training opportunities in Canada?
   - Yes
   - No

8. What kind of support in developing cross-cultural competence and language skills for IMGs would you like to see from government IMG support programs and IMG organizations?

Part IV. Current Occupational Status

1. Which of the following best describes your current occupational status?
   - Employed, full-time as a medical resident in Canada or the US
   - Employed, full-time other jobs
   - Employed, part-time
   - Not employed, but currently looking for a job
   - Not employed, not currently looking for a job

2. If you are employed, are you employed in a healthcare-related field?
   - Yes.
   - No.
   - Not applicable.

If you answer is yes, please specify the type of job:

3. Where did you get the employment opportunities for your current job?
   - Government IMG support programs
   - IMG organizations
   - Employment advertisement online
   - Family members or friends
   - Other, please specify:

4. How difficult do you feel it is to find a job in a healthcare-related field as an IMG? Please rate from 1 to 10 (1 represents least difficult, and 10 represents very difficult).

5. Which of the following range best describes your current annual salary?
   - No income
   - <10,000 CAD
   - 10,000-30,000 CAD
   - 30,000-50,000 CAD
   - 50,000-100,000 CAD
   - >100,000 CAD

6. Have you experienced any financial strain during your preparation for residency application in Canada?
   - Yes.
   - No.

7. Who is the main source of financial income for your family?
   - Myself

- Other members of the family

8. Is your family supportive of you to take MCC exams and prepare for residency application in Canada? Please rate from 1-10 (1 represents least supportive, and 10 represents very supportive) or choose Not Applicable.

Part V. Group Specific Opinions and Comments

If you are currently actively preparing for residency match, please go to **Section 1**.
If you are not actively pursuing a residency position now, but have tried in the past, please go to **Section 2**.
If you have successfully matched to a residency program in Canada or the US, please go to **Section 3**.

Section 1:
1. Are you also considering or preparing for an alternative career path in case you cannot get matched in Canada?
- o Definitely yes
- o Probably yes
- o Might or might not
- o Probably not
- o Definitely not

2. What kind of alternative career path are you considering or preparing for?
- o Physician assistant
- o Medical technologist
- o Nursing
- o Medical research
- o Medical management
- o Non-medical related field
- o Other, please specify:

3. Are you considering going back to your country of origin to practice in case you cannot get matched in Canada?
- o Yes
- o No
- o Not sure
- o Not applicable, I am a Canadian who studied medicine aboard

4. What do you think government IMG support agencies and local medical community can do to help IMGs find an alternative career path in the medical field?

Section 2:
1. What are the factors that made you stop pursuing residency training in Canada or the US? (Please choose all that apply)
- o Family responsibilities

- Financial strain
- Matched in countries other than Canada and the US
- Return to my country of origin to practice
- Other job opportunities
- Frustration
- Other, please specify:

2. How many times have you applied through the CaRMS?
- 0
- 1
- 2
- 3
- 4
- 5
- 6
- 7
- >7

3. What kind of additional training have you received for your current job?
- Not working
- Research fellowship
- Graduate study
- Professional training at a college
- Conferences
- Hospital/University open lectures/grand rounds
- Other, please specify

4. If you are currently working, are you satisfied with your job? Please rate your experience from 1-10 (1 represents lease satisfied, and 10 represents most satisfied)

5. Do you think you get enough support from government IMG support agencies and local medical community to find a job?
- True
- False

6. Are the MCC examinations and CaRMs experience helpful for you to find a job? Please rate your experience from 1-10 (1 represents lease helpful, and 10 represents most helpful)

7. Are you considering going back to your country of origin to practice?
- Yes

- No
- Not sure

7. What additional support do you want to see from government IMG support agencies and local medical community for people like you?

Section 3:

1. Have you matched to a residency program in Canada or the US?
   - Canada
   - USA

1. How many times did you apply through the CaMRS or the US version before you got your residency position in Canada or the US?
   - 0
   - 1
   - 2
   - 3
   - 4
   - 5
   - 6
   - 7
   - >7

2. How long was the gap between the start of your residency in Canada or the US and your last practice in your country of origin?
   - 1 year
   - 2 years
   - 3 years
   - 4 years
   - 5 years
   - 6 years
   - 7 years
   - 8 years
   - 9 years
   - 10 years
   - >10 years

3. What do you think is the most important factor or factors that helped you succeed in matching to a residency training program in Canada or the US? (Please choose all that apply)
   - Training background in another country (other than Canada and the US)

- High MCC exam scores
- Clinical fellowship in Canada
- Clinical fellowship in the US
- Clinical observership in Canada
- Clinical observership in the US
- Graduate studies in Canada
- Graduate studies in the US
- Research experience in Canada
- Research experience in the US
- Volunteer experience
- Other, please specify:

4. Are you satisfied with your residency training experience? Please rate your experience from 1-10 (1 represents lease satisfied, and 10 represents most satisfied)
Please give the reasons for your rating above:

5. Are you currently contributing to clinical medicine or medical education in your country of origin?
   - Yes
   - No

If yes, can you provide some details of the activities or projects?

6. Are you currently assisting other IMGs who are seeking residency positions?
   - Yes
   - No

If yes, can you provide some details of the activities or projects?

7. If given the opportunity, are you willing to assist other IMGs who are seeking residency positions?
   - Yes
   - No
   - Not sure

8. If you are matched to a US residency program, how long did it take for you to switch from seeking residency in Canada to residency in the US?
   - 1 year
   - 2 years
   - 3 years
   - 6 years
   - 5 years
   - > 5 years

9. What suggestions do you have for government IMG agencies and local medical community to support IMGs?
